# Reelin and Neuropsychiatric Disorders

**DOI:** 10.3389/fncel.2016.00229

**Published:** 2016-10-18

**Authors:** Kazuhiro Ishii, Ken-ichiro Kubo, Kazunori Nakajima

**Affiliations:** Department of Anatomy, Keio University School of MedicineTokyo, Japan

**Keywords:** reelin, psychiatric disorder, schizophrenia, animal model, *reeler*

## Abstract

Proper neuronal migration and laminar formation during corticogenesis is essential for normal brain function. Disruption of these developmental processes is thought to be involved in the pathogenesis of some neuropsychiatric conditions. Especially, Reelin, a glycoprotein mainly secreted by the Cajal-Retzius cells and a subpopulation of GABAergic interneurons, has been shown to play a critical role, both during embryonic and postnatal periods. Indeed, animal studies have clearly revealed that Reelin is an essential molecule for proper migration of cortical neurons and finally regulates the cell positioning in the cortex during embryonic and early postnatal stages; by contrast, Reelin signaling is closely involved in synaptic function in adulthood. In humans, genetic studies have shown that the *reelin* gene (*RELN*) is associated with a number of psychiatric diseases, including Schizophrenia (SZ), bipolar disorder (BP) and autistic spectrum disorder. Indeed, *Reln* haploinsufficiency has been shown to cause cognitive impairment in rodents, suggesting the expression level of the Reelin protein is closely related to the higher brain functions. However, the molecular abnormalities in the Reelin pathway involved in the pathogenesis of psychiatric disorders are not yet fully understood. In this article, we review the current progress in the understanding of the Reelin functions that could be related to the pathogenesis of psychiatric disorders. Furthermore, we discuss the basis for selecting Reelin and molecules in its downstream signaling pathway as potential therapeutic targets for psychiatric illnesses.

## Introduction

Falconer (1951) first described the mutant *reeler* mouse, which is characterized by reeling gait caused by dysregulation of motor coordination and ataxia. More than four decades later, the gene responsible for the *reeler* phenotype was identified and the protein encoded by the gene was named Reelin (Bar et al., 1995; D’Arcangelo et al., 1995; Hirotsune et al., 1995; Ogawa et al., 1995). Until date, much work has been carried out towards understanding the functions of Reelin during cortical development, because the Reelin-deficient mutant mouse, *reeler*, shows largely inverted cortical layers (Caviness and Sidman, 1973; Tissir and Goffinet, 2003; Honda et al., 2011; Sekine et al., 2014).

**Figure 1:**
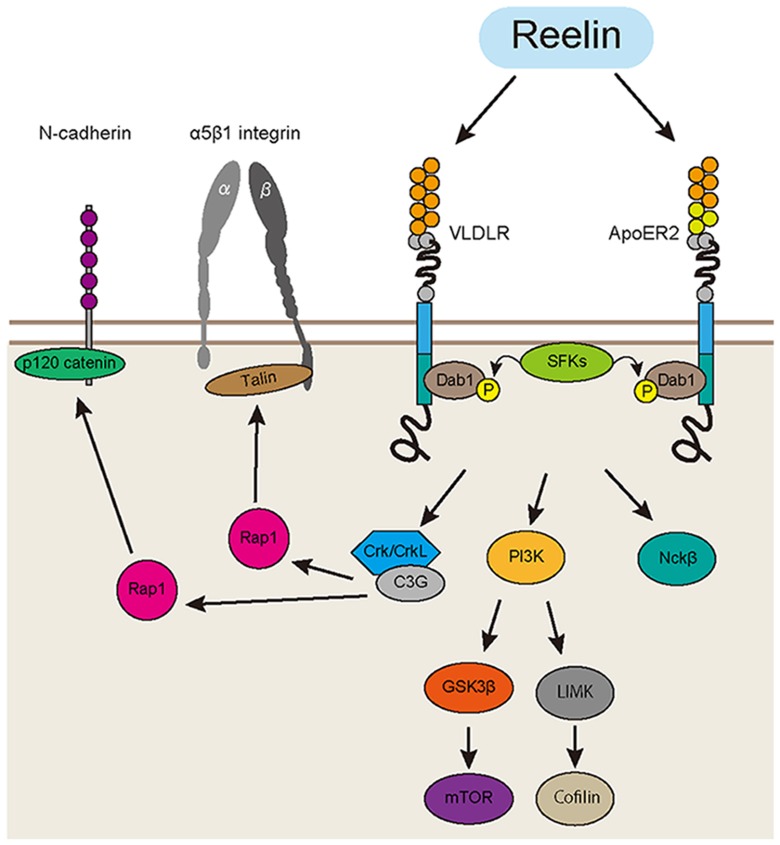
**Reelin signaling pathway in the developing cortex.** The Reelin protein binds to very-low-density lipoprotein receptor (VLDLR) and apolipoprotein E receptor 2 (ApoER2). Then, Dab1 is phosphorylated by the Src-family tyrosine kinases (SFKs). Phosphorylated Dab1 recruits downstream molecules such as Crk/CrkL, phosphatidylinositol 3-kinase (PI3K) and Nckβ. CrkL binds to C3G, an effector protein. C3G promotes the formation of Rap1-GTP, which activates cell adhesion molecules, including α5β1 integrin and N-cadherin. There are also Dab1-PI3K-mTOR, Dab1-PI3K-cofilin and Dab1-Nckβ pathways, which regulate the actin cytoskeleton relevant to dendrite formation and neuronal migration in the developing cortex.

Reelin is a glycoprotein secreted mainly from the Cajal-Retzius cells in the developing cerebral cortex and hippocampus, and acts as a key regulator of various aspects of laminar formation, including neuronal migration, cell aggregation and dendrite formation (Nakajima et al., 1997; Kubo et al., 2010; Franco et al., 2011; Jossin and Cooper, 2011; Sekine et al., 2014; Kohno et al., 2015). Many downstream molecules and several pathways involved in Reelin signaling during development have been elucidated from animal studies (Howell et al., 1997; Hiesberger et al., 1999; Ballif et al., 2004; Honda et al., 2011; Jossin and Cooper, 2011; Sekine et al., 2012; Figure 1).

**Figure 2:**
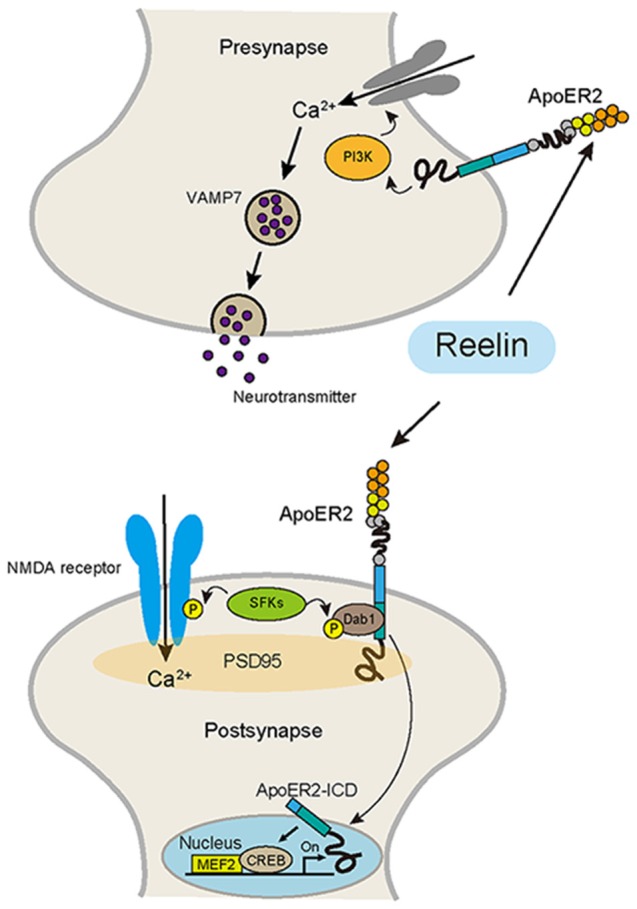
**Reelin signaling pathway in the adult brain, which regulates synaptic function.** Reelin binds to ApoER2 and activates SFKs, which phosphorylate a tyrosine of the NR2 subunit of the N-methyl-D-aspartate (NMDA) receptor, resulting in the potentiation of NMDA receptor-mediated Ca^2+^ influx. ApoER2 binds to PSD-95, a scaffolding protein in the post synapse. ApoER2 is proteolytically cleaved by γ-secretase activity, which is activated by NMDA receptor signaling. Then, the intracellular domain of ApoER2 (ApoER2-ICD) is released from the cell membrane and translocates into the nucleus and activates transcriptional factors, including MEF2 and CREB. In the presynapse, the Reelin-ApoER2-PI3K pathway promotes Ca^2+^ influx, which leads to the selective mobilization of VAMP7-enriched synaptic vesicles, promoting neurotransmitter release.

In the postnatal period, the distribution and expression patterns of Reelin are dramatically changed as compared to those during the embryonic period (Alcántara et al., 1998). This suggests that the roles of Reelin in the postnatal brain might also be changed. Intriguingly, a number of lines of evidence indicate that Reelin signaling modulates synaptic function in the adult brain (Herz and Chen, 2006; Figure 2).

In humans, genetic studies have reported that the *RELN* locus is associated with neuropsychiatric disorders, such as Schizophrenia (SZ), bipolar disorder (BP), autism spectrum disorder (ASD) and Alzheimer’s disease (AD; Ovadia and Shifman, 2011; Wang et al., 2014; Bufill et al., 2015; Li et al., 2015). Indeed, homozygous (*rl/rl*) and heterozygous *reeler* (*rl/-*) mice haploinsufficient for Reelin show cognitive and behavioral abnormalities (Tueting et al., 1999; Qiu et al., 2006a), supporting the notion that *RELN* haploinsufficiency may lead to higher brain dysfunctions relevant to neuropsychiatric disorders in humans. A number of studies have revealed that Reelin also plays a pivotal role in regulating synaptic functions, including N-methyl-d-aspartate (NMDA) receptor signaling (Beffert et al., 2005; Qiu et al., 2006b), and these findings may give a clue to uncover the impairments in the Reelin signaling pathway underlying the development of psychiatric disorders.

In this article, we first provide an overview of the current progress in Reelin research at the molecular, cellular and tissue levels. Second, we review human genetic studies of neuropsychiatric disorders associated with mutations in the *RELN* gene locus. Then, we focus on how Reelin dysfunction leads to the behavioral abnormalities relevant to neuropsychiatric diseases in mouse models. Finally, we discuss the basis for selecting Reelin and molecules in its downstream signaling pathway as potential therapeutic targets for neuropsychiatric disorders.

## Reelin has an Essential Role in Cortical Development

In mammals, cortical expansion is thought to contribute to the acquisition of higher brain functions during the course of evolution (Molnar et al., 2006). The neocortex is composed of a well-organized six-layer structure, including excitatory and inhibitory neurons (Rakic, 2009). The excitatory neurons, born directly or indirectly from the radial glia (Tabata et al., 2009; Sekine et al., 2013), which are neural progenitor cells located in the ventricular zone, migrate radially towards the brain surface, and eventually reach their final destinations (Rakic, 1972). Since the late-born neurons migrate past their predecessors, the earlier-born neurons are placed at a deeper position and the later-born neurons at a more superficial position in the cortical plate (CP; Caviness, 1982; Takahashi et al., 1999). This pattern of cell alignment is called a birth-date-dependent “inside-out” pattern. By contrast, inhibitory interneurons are born in the ganglionic eminences (GEs) in the ventral telencephalon and preoptic area in the rostral diencephalon that are located far from their final destination, and migrate tangentially for a long distance towards the dorsal pallium (Anderson et al., 1997; Tamamaki et al., 1997; Yozu et al., 2005; Kanatani et al., 2008, 2015; Gelman et al., 2009, 2011; Marin et al., 2010). Correct neuronal migration and laminar formation are essential for the establishment of proper brain functions. Indeed, disruption of the cortical architecture has been observed in various neuropsychiatric disorders (Arnold, 2000; Wegiel et al., 2010).

Reelin is a glycoprotein that is mainly secreted from the Cajal-Retzius cells located in the marginal zone (MZ), and a subpopulation of GABAergic interneurons (Rice and Curran, 2001). Reelin-deficient mice, *reeler*, show largely inverted cortical layers, which strongly supports the notion that Reelin is a key regulator of cortical development. A recent study showed that cortical laminar formation in *reeler* brains exhibit a more complex pattern than previously thought. Boyle et al. (2011) reported that the layer formation is severely disorganized in the *reeler* cortex, with layer II/III neurons located in the middle of the cortex and cells from other layers (layer IV, V and VI) split between the deep and superficial layers in a mirror image fashion. The secreted Reelin protein binds to its receptors, apolipoprotein E receptor 2 (ApoER2) and very-low-density lipoprotein receptor (VLDLR), which are mainly expressed on the cell membrane of cortical neurons (Trommsdorff et al., 1999; Hirota et al., 2015). Then, disabled homolog 1 (Dab1), an intracellular adaptor protein, is phosphorylated via Fyn and Src, the Src-family tyrosine kinases (SFKs; Hiesberger et al., 1999; Howell et al., 1999). Phosphorylated Dab1 recruits and binds with several downstream molecules, including Crk, phosphatidylinositol 3-kinase (PI3K), and Nckβ (Beffert et al., 2002; Pramatarova et al., 2003; Park and Curran, 2008). The Crk family proteins (Crk and CrkL) are adaptor proteins that bind to phosphorylated Dab1 and recruit their effector proteins (Chen et al., 2004). Park and Curran (2008) reported that conditional double-knockout mice of *Crk* and *CrkL* showed the major anatomic features of *reeler*, including disruption of layer formation in the cerebral cortex and hippocampus, absence of preplate splitting, impaired dendrite formation, and cerebellar hypofoliation. The brains of mice deficient in C3G, an effector protein of CrkL, also show impaired preplate splitting (Voss et al., 2008). These findings indicate the Reelin-Dab1-Crk/CrkL pathway plays a critical role in controlling layer formation in the cortex, including preplate splitting. Sekine et al. (2012) recently reported that Reelin activates integrin α5β1 through an intracellular Dab1-Crk/CrkL-C3G-Rap1 pathway. Furthermore, they showed that activated integrin α5β1 controls the terminal translocation, a final mode of neuronal movement beneath the MZ (Nadarajah et al., 2001; Sekine et al., 2011, 2012). Other groups reported that Reelin signaling regulates the function of another cell adhesion molecule, N-cadherin. Franco et al. (2011) reported that Dab1 acts on the migratory neurons to stabilize their leading processes in a Rap1-dependent manner. They showed that Rap1 regulates the function of N-cadherin and finally controls the somal translocation (Franco et al., 2011). The same group also reported that N-cadherin activated through the Reelin-Rap1 pathway enhances heterotypic cell-cell contact between the Cajar-Retzius cells and migratory neurons (Gil-Sanz et al., 2013). Jossin and Cooper (2011) demonstrated that the Rap1-N-cadherin pathway also regulates the transition of migratory neurons from multipolar cells to bipolar cells beneath the CP; this mode change is crucial for the migratory neurons to enter the CP (Figure 1).

Reelin signaling plays a role in the processes of dendrite development (Olson et al., 2006; Jossin and Goffinet, 2007). Olson et al. (2006) found that Dab1-suppressed migrating neurons showed simplified leading processes that were less likely to attach to the MZ and exhibited abnormal cell positioning. Jossin and Goffinet (2007) reported that Reelin promoted dendritogenesis through activation of mammalian target of rapamycin (mTOR) and S6 kinase 1 (S6K1) in a Dab1-, PI3K- and Akt-dependent manner. In regard to axon formation, by ablation of Cajal-Retzius cells in a slice and analysis of *reeler* mice, Del Rio et al. (1997) demonstrated abnormalities in the development of axon fibers from the entorhinal cortex to the hippocampus. However, using *reeler* cortical explants, Jossin and Goffinet (2001) found that Reelin itself did not exhibit any significant attraction or repulsion to cortical axons.

In the adult brain, the main source of Reelin is no longer the Cajal-Retzius cells, but a subpopulation of GABAergic interneurons, a change that is also associated with a change in the distribution pattern of Reelin (Alcántara et al., 1998; Pesold et al., 1998). These findings suggest that the roles of Reelin in the adult brain may also be different from those in the developing brain. We shall review and discuss the functions of Reelin in the adult brain in “Reelin Regulation of Brain Function and Behavior” Section.

## Genetic Study of *RELN* in Neuropsychiatric Disorders

In humans, the mutations of the *RELN* have been shown to be associated with autosomal recessive lissencephaly with cerebellar hypoplasia (Hong et al., 2000). The brain phenotypes in these patients are similar to those found in the *reeler* mice, including abnormal laminar formation and cerebellar hypoplasia. In addition to this severe brain malformation, mutations in the *RELN* locus are also associated with neuropsychiatric disorders, with no apparent abnormalities in the brain structure. In this section, we provide an overview of the recent genetic studies carried out to examine the association of *RELN* with neuropsychiatric disorders.

### Schizophrenia

SZ is a devastating psychiatric disease that affects approximately 1% of the population and is characterized by hallucinations, delusions and cognitive disturbances. The first clinical features of SZ typically emerge between early childhood and adolescence, with many patients experiencing chronic SZ symptoms (Sawa and Snyder, 2002). The clinical symptoms are based on brain dysfunctions attributed to genetic and environmental factors (Insel, 2010). As SZ shows high heritability, the risk genes with the greatest impact on the predisposition to SZ have been pursued by many researchers (Schizophrenia Working Group of the Psychiatric Genomics Consortium, 2014). As for the *RELN* gene, Li et al. (2013) conducted a case-control association study and identified six single-nucleotide polymorphisms (SNPs; rs2237628, rs362626, rs362814, rs362813, rs362731, rs362726) which are located in the intron 29 of the *RELN* gene that were significantly associated with the risk of SZ in the Chinese population. Several groups have reported that the existence of a gender-specific (women) association between SNPs in *RELN* and SZ (Shifman et al., 2008; Kuang et al., 2011; Li et al., 2011). Li et al. (2011) also reported the association of another SNP (rs12705169) in the *RELN* locus with SZ. Although rs12705169 is located in the intron of *RELN* and does not lead to any change of the protein structure or function, it may be involved in alternative RNA splicing and miRNA generation. When they divided the subjects by gender, they found that this SNP was positively associated with SZ only in women. Similarly, Kuang et al. (2011) demonstrated an association of rs362719 of *RELN*, which is located in the exon 42 and contributes to the EGF-like domain of the fifth Reelin repeat, with susceptibility to SZ in their female, but not male, participants. Shifman et al. (2008) performed a genome-wide association study (GWAS) for SZ in Ashkenazi Jews and showed that rs7341475, a SNP in the intron four of the *RELN*, was associated with SZ only in women. However, the association of this SNP (rs7341475) with SZ was not replicated in any other study in the Chinese population (Liu et al., 2011). Subsequently, Ben-David and Shifman (2010) performed a meta-analysis of four GWAS studies to assess the association of rs7341475 with SZ, and suggested this locus might have a small effect on the SZ risk. Therefore, the association of rs7341475 with SZ may remain inconclusive. Wedenoja et al. (2008) replicated the previous linkage of SZ on 7q22 in an independent subject. They also studied four candidate genes, including *RELN*. Although the selected candidate genes showed no association to the clinical diagnosis of SZ, the quantifiable trait component analysis revealed that allelic variants of *RELN* contributed to the endophenotypes of SZ, including working memory and executive function (Wedenoja et al., 2008).

### Other Neuropsychiatric Disorders and Neurological Diseases

ASD is characterized by impairments of social interaction and communication, as well as repetitive behaviors and restricted interests (Maenner et al., 2014). Twin and family studies have revealed the important role of genetic factors in ASD, with a heritability value of as high as 90% (Freitag et al., 2007). Although many studies have been conducted to explore the genetic association between *RELN* and ASD, the results have been inconclusive. Persico et al. (2001) was the first to report that polymorphic GGC repeats located in the 5′ untranslated region (5′ UTR) of the *RELN* were associated with autistic disorder, finding that was subsequently replicated in three studies (Zhang et al., 2002; Skaar et al., 2005; Dutta et al., 2007). However, other groups have failed to show an association between the triplet repeats in the 5′ UTR of the *RELN* and autism (Krebs et al., 2002; Bonora et al., 2003; Devlin et al., 2004; Li et al., 2004). In the family-based association analyses carried out by Serajee et al. (2006), the most significant results were the apparent association of autism, in a broad diagnosis of the disease, with rs736707 in intron 59 and rs362691 in exon 22 of the *RELN*. Li et al. (2008) also showed a significant genetic association between rs736707 in intron 59 and ASD in a Han Chinese population. On the other hand, other groups have failed to detect these associations (Dutta et al., 2008; He et al., 2011). Dutta et al. (2008) carried out case-control and family-based association studies for six SNPs (rs727531, rs2072403, rs2072402, rs362691, rs362719, rs736707), and found that these SNPs of *RELN* were unlikely to be associated with ASD. Recently, a Chinese group conducted a meta-analysis for case-control and transmission disequilibrium test (TDT) studies published from 2001 to 2013 and concluded that rs362691 might contribute significantly to the risk of ASD (Wang et al., 2014).

There are only a few reports that suggested the existence of an association between the* RELN* and BP (Goes et al., 2010; Ovadia and Shifman, 2011). Goes et al. (2010) found that the rs362719 of *RELN* was associated with susceptibility to BP, particularly in females.

Several groups have also reported an association between AD and the *RELN* gene (Seripa et al., 2008; Antoniades et al., 2011; Kramer et al., 2011; Bufill et al., 2015; Fehér et al., 2015). AD is a neurodegenerative disease characterized by the formation of neurofibrillary tangles and beta-amyloid plaques in the brain, and patients clinically manifest progressive impairments of memory and cognition (Scheltens et al., 2016). Several SNPs in the *RELN* gene have been reported to be associated with the risk of AD. Seripa et al. (2008) showed an association between a triplet tandem repeat in the 5′UTR and rs607755 with AD, particularly in females. Another group also showed significant association between rs607755 and the risk of AD (Fehér et al., 2015). However, their results were inconsistent with the conclusion by Seripa et al. (2008) that rs607755 was significantly associated with AD only in males (Fehér et al., 2015). Antoniades et al. (2011) reported that an SNP in exon 22 of *RELN* (rs362691) was significantly associated with the risk of AD in a Greek population. Bufill et al. (2015) reported that SNPs in the *RELN* gene (rs528528 and rs2299356) and two genes (*PLK2* and *CAMK2A*) related to the Reelin signaling pathway were associated with AD and mild cognitive impairment (MCI).

## Reelin Regulation of Brain Function and Behavior

### Expression Level of Reelin and Vulnerability to Neuropsychiatric Disorders

Many studies have provided evidence for altered Reelin expression in rodents with cognitive dysfunction, which may relate to neuropsychiatric diseases. Heterozygous *reeler* mice (HRM), in which the amount of Reelin protein is approximately 50% as compared to that in the wild type mice, exhibit behavioral abnormalities (Costa et al., 2002). Thus, HRM has been of interest as an animal model of psychiatric diseases. In spite of the initial inconsistent findings on the occurrence of behavioral deficits in HRM (Salinger et al., 2003; Podhorna and Didriksen, 2004), many groups have reported behavioral traits associated with *Reln* haploinsufficiency in HRM (Tueting et al., 1999; Qiu et al., 2006a; Barr et al., 2007, 2008; Teixeira et al., 2011; Kutiyanawalla et al., 2012; Iafrati et al., 2014). Several groups have also reported defect in the prepulse inhibition (PPI), an impairment of sensory motor gating that is associated with SZ, in HRM (Tueting et al., 1999; Barr et al., 2008; Teixeira et al., 2011; Kutiyanawalla et al., 2012). Iafrati et al. (2014) showed that Reelin deficiency in HRM caused defects in the juvenile morphofunctional properties of excitatory synapses in the prefrontal cortex (PFC) and behavioral dysfunction in prefrontal circuits. Furthermore, Barr et al. (2007) showed that the Reelin receptors VLDLR and ApoER2 regulated sensory motor gating. They demonstrated that acoustic PPI was intact in both *Vldlr*- and *Apoer2*-mutant mice. However, *Vldlr*-homozygous knockouts mice exhibited deficits in crossmodal PPI, while *Apoer2*-heterozygous and homozygous knockouts mice exhibited increased crossmodal PPI (Barr et al., 2007). Qiu et al. (2006a) demonstrated that HRM exhibited hippocampus-dependent learning deficit underlying the impairment of hippocampal plasticity. There are several interesting studies to show that mouse behavioral alterations are manifested as a consequence of gene-environment interaction, similar to many psychiatric disorders in humans (Laviola et al., 2009; Romano et al., 2014; Howell and Pillai, 2016). There have also been a number of reports indicating that in humans, the amount of Reelin protein in the brain, plasma and cerebrospinal fluid (CSF) are associated with neuropsychiatric conditions. Almost all studies have shown that the reduced amount of Reelin in the brain and blood are associated with a high risk of development of neuropsychiatric disorders (Guidotti et al., 2000; Fatemi et al., 2005; Eastwood and Harrison, 2006). Interestingly, however, increased expression levels of Reelin in the CSF (Sáez-Valero et al., 2003) and frontal cortex (Botella-López et al., 2006) were found in case of AD. Accumulation of Reelin has also been reported in amyloid-like plaques, with a decline in Reelin-positive neurons, which might represent a risk factor for AD (Knuesel et al., 2009). Reelin protein itself is thought to be protective against AD-like neuropathology, since the reduced Reelin expression in a transgenic AD background markedly elevated amyloid-β plaque formation (Kocherhans et al., 2010).

### The Expression Level of Reelin is Regulated by Multiple Mechanisms

In addition to genetic mutations/polymorphisms, various mechanisms may lead to aberrant expression of Reelin. In the postnatal brain, the main source of Reelin is shifted from Cajal-Retzius cells to a subpopulation of GABAergic interneurons (Alcántara et al., 1998; Pesold et al., 1998). Using *in situ* hybridization analysis combined with immunohistochemistry, Pesold et al. (1998) showed that Reelin is preferentially expressed in GABAergic interneurons in the adult rat cortex and hippocampus. Furthermore, they found Reelin immunoreactivity not only in neurons, but also in the extracellular space, suggesting that the GABAergic interneurons secrete Reelin into the extracellular matrix (Pesold et al., 1998). However, how Reelin is secreted by these specialized interneurons in the postnatal brain remains an unresolved question, although a constitutive mechanism dependent on a specific sequence of positively charged amino acids in the Reelin carboxy terminus domain has been reported (Rodriguez et al., 2000). Thus, in mature brains, the amount of secreted Reelin protein may depend on the number and distribution of Reelin-positive interneurons.

Several studies have shown the involvement of epigenetic mechanisms in the transcriptional regulation of *RELN* (Abdolmaleky et al., 2005; Grayson et al., 2005). Grayson et al. (2005) analyzed the pattern of methylation within the CpG island of the *RELN* promoter in human SZ brains obtained from a brain bank. They showed hypermethylation of the promoter region of *RELN* in the SZ brains (Grayson et al., 2005). Abdolmaleky et al. (2005) also demonstrated hypermethylation of a CpG island containing CRE and an SP1-binding site in the promoter region of *RELN* in post-mortem examination of the brains of SZ patients. However, Tochigi et al. (2008) reanalyzed this *RELN* promoter region by using the same brain samples as in the previous study, and showed that the extent of methylation in this promoter region did not differ significantly between the SZ patients and controls. Furthermore, Mill et al. (2008) prepared a microarray-based comprehensive epigenomic scan and found epigenetic changes in the loci associated with glutamatergic and GABAergic neurotransmitter pathways. However, they did not detect any association between hypermethylation in the promoter region of *RELN* and major psychosis. Recently, a systematic review of DNA methylation in SZ and BP has been reported (Teroganova et al., 2016). Although a number of differentially methylated genes were detected, including *RELN*, diverse methodologies used across studies hampered the reliability of the meta-analysis (Teroganova et al., 2016). Thus, in regard to the epigenetic regulation of *RELN* expression, further investigation is needed to confirm the association of hypermethylation in the *RELN* promoter region with the predisposition to major mental illnesses.

Perturbation of transcriptional regulation could affect the Reelin protein distribution as well as expression levels. Baek et al. (2015) reported that *RELN* transcription was derepressed in ectopic cortical regions, mediated by activation of the transcription factor FOXG1 in human focal cortical malformations. They found that misexpression of Reelin in the progenitor cells caused non-cell autonomous neuronal migration defects, resulting in focal cortical malformations. These results may, at least to some extent, resemble the effects of ectopic Reelin overexpression in the mouse brain (Kubo et al., 2010).

### Reelin Signaling is Involved in Synaptic Functions and Behavior

During cortical development, the main source of Reelin is the Cajal-Retzius cells located in the MZ. Subsequently, these cells gradually disappeared, and the main source of Reelin finally shifts to a subtype of GABAergic interneurons (Miyoshi et al., 2010). Thus, during this period, the expression pattern of Reelin changes dynamically. This suggests that the role of Reelin in the postnatal brain also changes dramatically. Indeed, in adulthood, there is much evidence to indicate that Reelin modulates NMDA receptor-mediated synaptic functions (Beffert et al., 2005; Iafrati et al., 2014; Figure 2). Dysfunction of NMDA receptor signaling plays a key role in the pathogenesis of major neuropsychiatric disorders, including SZ and AD (Zhou and Sheng, 2013). Recently, it has been reported that hundreds of genetic loci were associated with SZ in a large-scale GWAS (Schizophrenia Working Group of the Psychiatric Genomics Consortium, 2014). In this analysis, the glutamatergic neurotransmission-related genes including *GRIN2A*, a NMDA receptor subunit, were highly associated with SZ. NMDA dysfunction has also been reported to be related to cognitive impairment in AD. In addition to the widely used drug, cholinesterase inhibitors, for AD, a new drug that modulates glutamate signaling has also become available recently for AD (Scarpini et al., 2003). Also, it has been shown that Reelin acts on NMDA receptor signaling via ApoER2, a Reelin receptor (Beffert et al., 2005; Chen et al., 2005; Herz and Chen, 2006). Beffert et al. (2005) demonstrated that an alternative splicing variant of ApoER2 modulated the NMDA receptor activity through Reelin signaling. They found that alternatively spliced exon 19, which encodes amino acids in the intracellular domain of ApoER2 (ApoER2-ICD), is necessary for the stimulation of tyrosine phosphorylation of the NMDA receptor subunit NR2 in hippocampal slices in response to Reelin. Furthermore, they confirmed that the mice expressing a mutant ApoER2 lacking exon 19 showed severe cognitive disturbances related to the NMDA receptor function (Beffert et al., 2005). Interestingly, birds do not have exon 19 (Brandes et al., 1997), suggesting that adding this new exon may partly contribute to the acquisition of higher brain functions in mammals. Chen et al. (2010) further showed that the Reelin-ApoER2-NMDA receptor pathway is involved in the pathophysiology of AD. They demonstrated that ApoE4, which is associated with an earlier average age at onset of dementia, depletes ApoER2 from the neuronal surface after ligand-induced endocytosis by Reelin, which impairs synaptic plasticity and NMDA phosphorylation induced by Reelin (Chen et al., 2010). Moreover, Lane-Donovan et al. (2015) demonstrated that Reelin signaling protects against the Aβ toxicity that induces synaptic dysfunction in the early stages of aging. Using Reelin conditional knockout mice, they found that the loss of Reelin rendered excitatory synapses susceptible to functional suppression by Aβ, which resulted in impaired learning and memory. Iafrati et al. (2014) demonstrated that *in vivo* injection with ketamine, an NMDA receptor antagonist, or Ro25-6981, an inhibitor of GluN2B-NMDA receptors, in the juvenile period rescued the behavioral abnormalities, reduced the dendritic spine density, and anomalous long-term potentiation (LTP) in the PFC of HRM. Their results suggest Reelin is essential for proper functional and behavioral development of juvenile prefrontal circuits through modulating the NMDAR-mediated signaling pathway.

Reelin signaling is also involved in the presynaptic functions (Figure 2). Reelin acts presynaptically in mature neurons to rapidly enhance neurotransmitter release. This role of Reelin depends on the function of VAMP7, a vesicular SNARE protein (Bal et al., 2013). Telese et al. (2015) reported interesting findings; they reported that the Reelin pathway controls learning and memory through activation of the transcriptional factors. Proteolytic cleavage of ApoER2 is a crucial component of the synapse-to-nuclear signaling triggered by Reelin. When Reelin binds to its receptor ApoER2, nuclear translocation of the ApoER2-ICD is triggered. Then, ApoER2-ICD binds to the transcriptional factors MEF2 and CREB, and as a result, expressions of some genes involved in synaptic plasticity are activated (Telese et al., 2015).

Observations in animal studies using *reeler* and Reelin receptor mutant mice support the notion that Reelin-synapse dysfunction leads to cognitive dysfunction and neuropsychiatric symptoms (Weeber et al., 2002; Qiu et al., 2006a,b; Pujadas et al., 2010; Trotter et al., 2013). Several groups have reported that Reelin plays an important role in synaptic plasticity, mainly dependent on the NMDA receptor dysfunction in the hippocampal region (Weeber et al., 2002; Qiu et al., 2006b; Pujadas et al., 2010). Weeber et al. (2002) reported that Reelin signaling is crucial for memory formation and synaptic plasticity in the hippocampal CA1 region. They demonstrated using hippocampal slices, that mice with KO of each of the Reelin receptors, VLDLR and ApoER2, exhibited defects in LTP. They further showed that treatment of the hippocampal slices obtained from wild-type mice with Reelin significantly enhanced the LTP in the CA1 region, which was abolished in mice deficient in either of the receptors. Qiu et al. (2006b) replicated *Weeber*’s findings by using HRM. They also observed defect of LTP in the CA1 region by electrophysiological analysis. In addition, according to a report by Pujadas et al. (2010), overexpression of Reelin enhanced the synaptic function in the hippocampus. They generated transgenic mice that overexpressed Reelin under the control of the CaMKIIα promoter. The mice showing Reelin overexpression exhibited increased spine hypertrophy and LTP in the hippocampus (Pujadas et al., 2010). Trotter et al. (2013) generated* Dab1*-conditional knockout mice and examined the spine morphology and synaptic functions. They conditionally deleted Dab1 protein from the excitatory neurons of the adult forebrain using *Dab1^flox/flox^*; *CaMKII-Cre* mice. This Cre driver line exhibits Cre expression in the forebrain, starting at approximately P19. They showed that loss of Dab1 led to a reduction of the spine size, suppression of Akt and ERK signaling, loss of hippocampal LTP, and deficits in hippocampus-dependent learning and memory. These results obtained using transgenic mice are consistent with the previous results obtained using *reeler* mice (Pujadas et al., 2010; Trotter et al., 2013). Furthermore, recently, Imai et al. (2016) generated dorsal forebrain-specific *Dab1* conditional knockout mice (*Dab1^flox/flox^*; *Emx1-Cre* mice), in which Cre is expressed specifically in the dorsal forebrain from the beginning of corticogenesis, and performed behavioral tests. These *Dab1*-conditional knockout mice showed normal motor functions, but exhibited hyperactivity, decreased anxiety-like behavior, and deficits in spatial reference and working memory (Imai et al., 2016). In these mice, in addition to the deficient Reelin-Dab1 signaling, the disorganized laminar structure in the cerebral cortex might also have contributed to the observed behavioral abnormalities. The best way to understand Reelin function in adulthood is to analyze conditional *Reelin* gene (*Reln*) knockout mice. Lane-Donovan et al. (2015) generated inducible conditional *Reln* knockout mice and analyzed them. After Reelin inactivation at 2 months of age, they found that while the mice had no apparent abnormalities of the cortical structure, they did exhibit mild behavioral changes and electrophysiological phenotype. Importantly, the behavioral phenotypes in the conditional *Reln* knockout mice may be somewhat different from those observed in *Reln*-deficienct (homozygous and heterozygous *reeler*) mice, which may be based on the degree of structural abnormalities. In the mature brain, the structure would not be seriously affected by Reelin deficiency. By contrast, Reelin deficiency during the developmental and early postnatal stages would severely affect the brain structure, which may be related to the development of some types of young-onset mental disorders. Further investigations in conditional *Reln* knockout mice are needed to clarify this issue.

In humans, dysfunction of the PFC, which plays crucial roles in cognitive functions, has been implicated in many psychiatric disorders (Weinberger et al., 1988). Analogous to the human PFC, the medial PFC (mPFC) is involved in various cognitive functions and social behaviors in rodents (Ishii et al., 2015a). Brosda et al. (2011) demonstrated that Reelin knockdown in the mPFC resulted in behavioral alterations in young adult rats, including disruption of sensorimotor gaiting and deficits in spatial working memory as well as object recognition. Sui et al. (2012) also reported that the expression level of Reelin was related to the regulation of LTP in the rat mPFC. They found that epigenetic regulation of *Reln* was involved in the induction of LTP in the mPFC, which resulted in behavioral alterations. Iafrati et al. (2014) also reported that HRM showed reduced dendritic spine density and abnormal LTP in the PFC. Interestingly, Ishii et al. (2015b) demonstrated dominant expression of VLDLR in both excitatory pyramidal neurons and GABAergic inhibitory interneurons, except for the migrating interneurons in the rostral migratory stream in the postnatal mPFC. As compared to the findings in the hippocampus, there is not much evidence to support the importance of Reelin signaling in the synaptic functions in the mPFC. Further investigations are needed to elucidate the role of Reelin in the functions of the mPFC relevant to mental illnesses.

## Reelin and its Downstream Signaling Molecules as a Target of Therapeutic Intervention

The findings described in the above sections suggest that Reelin pathways could be potentially useful as targets of therapeutic interventions for neuropsychiatric disorders. Indeed, several groups have directly assessed this possibility by examining the effects of administration of Reelin protein into the mouse brain (Rogers et al., 2011, 2013; Hethorn et al., 2015; Ishii et al., 2015b). Ishii et al. (2015b) demonstrated that Reelin exerted a preventive effect on phencyclidine (PCP)-induced behavioral deficits. They injected a conditioned medium containing Reelin protein into the mouse cerebral ventricles before administering PCP, and assessed the behavior of the animals. The group of mice that had received prior administration of Reelin showed normal cognitive and sensory-motor gating, indicating that the PCP-induced brain dysfunction was prevented by the Reelin injection. This study is based on their previous findings that prior transplantation of GABAergic neuronal progenitors into the mPFC of mice prevented the behavioral deficits induced by PCP and that the transplanted progenitors preferentially differentiated into Reelin/somatostatin-double positive GABAergic neurons specifically in the mPFC (Tanaka et al., 2011). Another group demonstrated that *in vivo* injection of Reelin into the mouse cerebral ventricle affected the synaptic functions and cognitive functions in wild-type mice and HRM (Rogers et al., 2011, 2013). The same group further showed that Reelin administration ameliorated both the synaptic plasticity and the cognitive behavioral deficits in a mouse model of Angelman syndrome, which is characterized by mental retardation, absence of speech, seizures and motor dysfunction (Hethorn et al., 2015).

Although we cannot directly apply this strategy of directly injecting Reelin into the human brain, modulating the activities of the signaling molecules downstream of Reelin could be a potential therapeutic approach. Reelin has been shown to activate PI3K and Akt (protein kinase B), to inhibit glycogen synthase kinase 3β (GSK3 β; Beffert et al., 2002) and activate the mTOR-S6K1 pathway (Jossin and Goffinet, 2007). Among them, GSK3 is already known as a target for mood stabilizers (De Sarno et al., 2002) and antipsychotics (Emamian et al., 2004; Li et al., 2007). In addition, animal models with disrupted mTOR signaling exhibit cognitive and behavioral deficits mimicking neuropsychiatric symptoms (Gururajan and van den Buuse, 2014). On the other hand, rapamycin, a well-established mTORC1 inhibitor, has been demonstrated to rescue cognitive impairments in several neurodevelopmental models of neuropsychiatric disorders, such as those of serotonin receptor (5-HT_6_) activation, neonatal PCP treatment, and post-weaning social isolation (Meffre et al., 2012), indicating overstimulation of mTOR signaling in these models. Surprisingly, rapamycin treatment also rescues lamination defects in tuberous sclerosis complex (TSC) 2-deficient mice, in which loss of TSC2 leads to activation of mTOR signaling and aberrant regulation of Reelin-Dab1 signaling (Moon et al., 2015). This inconsistency in the mTOR activities between different models might be explained by the varied activities of mTOR signaling as well as Reelin signaling among different brain regions and/or neuronal populations. Since such variation of Reelin (and its downstream) signaling activities could underlie the complex behavioral phenotypes of human neuropsychiatric disorders, further studies are required to distinguish clinical phenotypes and/or subpopulations that reflect disrupted Reelin signaling.

## Conclusion

In the developing cortex, Reelin is a key regulator of neuronal migration and laminar formation, which are essential for achieving higher brain functions in mammals. Intriguingly, however, the expression pattern of Reelin and its functions are dramatically different in the postnatal brain as compared to the embryonic brain. Indeed, Reelin modulates synaptic functions, which may also be based on the structural changes of the dendrites and spines in the postnatal period, which are closely related to cognitive behaviors and predisposition to neuropsychiatric symptoms. Although genetic studies have lent support to the notion of an association between the *RELN* gene and neuropsychiatric disorders, recent large-scale genome-wide analyses have revealed that the contribution of *RELN* mutations/polymorphisms to the development of neuropsychiatric disorders is not much higher than previously thought. However, many studies have indicated multiple mechanisms underlying the regulation of Reelin production, e.g., epigenetic regulation, cleavage events, and subtype specification of GABAergic interneurons. Perturbation of these regulatory mechanisms can also lead to brain dysfunction. The findings of animal experiments suggest that Reelin and its downstream signaling are closely related to the synaptic functions that underlie the mouse behaviors relevant to neuropsychiatric disorders. Therefore, the Reelin pathway is a potential therapeutic intervention target for neuropsychiatric disorders. Further investigations are needed to clarify how Reelin signaling regulates the higher brain functions and how it is involved in the development of neuropsychiatric disorders.

## Author Contributions

All authors contributed equally to the ideas and editing of the manuscript.

## Conflict of Interest Statement

The authors declare that the research was conducted in the absence of any commercial or financial relationships that could be construed as a potential conflict of interest.
